# Medical ozone: mechanisms, biological effects and clinical evidence—a narrative review

**DOI:** 10.3389/fmed.2026.1859221

**Published:** 2026-05-28

**Authors:** Danuta Lietz-Kijak, Karolina Skibicka, Piotr Skomro, Andrzej Brodkiewicz, Marta Budkowska, Krystiana Kijak, Helena Gronwald, Karina Kijak, Jan Szczegielniak, Katarzyna Bogacz, Aleksandra Bitenc-Jasiejko, Lidia Szczucka, Adam Andrzej Garstka, Małgorzata Wójcik

**Affiliations:** 1 Department of Propaedeutic, Physical Diagnostics and Dental Physiotherapy, Faculty of Medicine and Dentistry, Pomeranian Medical University, Szczecin, Poland; 2 Brodkiewicz & Skibicka Medical Center, Szczecin, Poland; 3 Department of Pediatrics, Child Nephrology, Dialysotherapy and Management of Acute Poisoning, Pomeranian Medical University, Szczecin, Poland; 4 Department of Medical Analytics, Pomeranian Medical University, Szczecin, Poland; 5 Student Scientific Society, Faculty of Medicine, Medical University of Białystok, Bialystok, Poland; 6 Student Scientific Society, Faculty of Medicine, Pomeranian Medical University, Szczecin, Poland; 7 Department of Physiotherapy, Faculty of Physical Education and Physiotherapy, Opole University of Technology, Opole, Poland; 8 Ministry of Internal Affairs and Administration’s Specialist Hospital of St. John Paul II, Glucholazy, Poland; 9 Department of Physiotherapy, Poznan University of Physical Education, Faculty of Sport Sciences in Gorzów Wlkp., Poznan, Poland

**Keywords:** adjunctive treatment, anti-inflammatory therapy, antimicrobial effect, clinical applications, medical ozone, Nrf2, oxidative stress, oxygen–ozone gas mixture

## Abstract

Medical ozone therapy has been increasingly explored as a potential adjunctive intervention across a range of clinical conditions. This narrative review aims to provide a critical overview of the biological mechanisms, biochemical interactions, and clinical applications of ozone therapy, with particular emphasis on the translational gap between experimental findings and clinical evidence. Ozone exerts its biological effects primarily through the generation of reactive oxygen species (ROS) and lipid ozonation products (LOPs), which act as secondary messengers influencing redox balance, immune responses, inflammation, and cellular metabolism. These mechanisms have been extensively characterized *in vitro* and in preclinical studies. However, their direct translation into clinically meaningful outcomes remains uncertain. Available clinical data suggest potential benefits of ozone therapy in selected contexts, including wound healing, musculoskeletal disorders, and infection control. Nevertheless, the majority of studies are small, heterogeneous, and often lack rigorous methodological design. High-quality randomized controlled trials are limited, and the overall level of evidence remains low to moderate depending on the indication. Recent systematic reviews and meta-analyses further emphasize the heterogeneity and methodological limitations of the available literature. Importantly, ozone therapy remains controversial within evidence-based medicine, with variability in regulatory acceptance across countries and limited endorsement from major health authorities. Safety data are also constrained by a lack of long-term studies and standardized treatment protocols. In conclusion, while ozone therapy demonstrates biologically plausible mechanisms and promising experimental effects, its clinical efficacy has not been conclusively established. At present, ozone therapy should be regarded as an investigational and adjunctive approach rather than a validated therapeutic modality, and further well-designed clinical trials are required to define its role in clinical practice.

## 1 Introduction

Ozone (O_3_) is a triatomic oxygen molecule with strong oxidative properties that has been explored for medical use in a variety of clinical contexts. Medical ozone is generated from pure oxygen and administered in controlled concentrations, typically as part of an oxygen–ozone gas mixture. Its proposed biological effects are primarily attributed to rapid reactions with biological fluids, leading to the formation of reactive oxygen species (ROS) and lipid ozonation products (LOPs), which act as secondary mediators influencing cellular signaling pathways (1–4). At the experimental level, ozone-induced oxidative signaling has been associated with modulation of redox homeostasis, activation of antioxidant defense systems, and alterations in immune and inflammatory responses. These mechanisms have been widely described *in vitro* and in preclinical studies, including the involvement of redox-sensitive pathways such as nuclear factor erythroid 2–related factor 2 (Nrf2) and nuclear factor kappa B (NF-κB) (5–7). More recent hypotheses also suggest that ozone or ozone-derived species may interact more directly with cellular signaling systems, including transient receptor potential (TRP) channels (8). In addition, emerging evidence indicates that ozone or ozone-related reactive species may be generated endogenously as part of physiological redox processes. This concept challenges the traditional view of ozone as exclusively exogenous and suggests a more complex role of ozone-related chemistry in cellular signaling and oxidative regulation (9). However, the biological and clinical relevance of endogenous ozone remains under investigation. Despite these mechanistic insights, the clinical relevance of ozone therapy remains uncertain. Most available evidence is derived from experimental models, small clinical studies, or observational reports with significant methodological heterogeneity (10–12). High-quality randomized controlled trials are limited, and the translation of proposed biological effects into consistent clinical outcomes has not been clearly established. Recent systematic reviews and meta-analyses further highlight the heterogeneity and generally low to moderate quality of evidence across different clinical indications (6–14). Despite its increasing use in some countries, ozone therapy remains controversial within evidence-based medicine. Major regulatory agencies, including the U.S. Food and Drug Administration (FDA), have not approved ozone for medical treatment due to insufficient evidence of safety and efficacy (15). In contrast, ozone therapy is practiced and regulated in several countries, including Italy, Spain, and parts of Latin America, under specific clinical frameworks. This variability in regulatory acceptance reflects ongoing uncertainty regarding its clinical value and underscores the need for critical evaluation of available evidence (16). Additional concerns include the potential for oxidative toxicity, the absence of standardized treatment protocols, and the lack of long-term safety data. Although ozone has been proposed as a therapeutic agent with pleiotropic effects such as antimicrobial (including antiviral) immunomodulatory, and anti-inflammatory actions these claims are often based on experimental findings that may not directly translate into clinical benefit. Given these limitations, a balanced and critical assessment of ozone therapy is warranted. This narrative review aims to synthesize current knowledge on the mechanisms of action, biochemical effects, and clinical applications of medical ozone, while explicitly addressing the limitations of the existing evidence base. Particular emphasis is placed on distinguishing between experimentally observed effects and clinically validated outcomes, as well as identifying key gaps for future resea.

## 2 Aim of the study

The aim of this narrative review is to provide a critical and structured evaluation of the current evidence on medical ozone therapy, with particular emphasis on the relationship between experimentally described mechanisms and their clinical relevance. Specifically, this review seeks to:

(1) Critically assess the proposed biological and biochemical mechanisms of ozone action, distinguishing between experimental findings and clinically validated effects;
(2) Evaluate the available clinical evidence across different indications, with attention to study design, methodological quality, and level of evidence;
(3) Analyze the variability in ozone administration methods and its impact on the interpretation of clinical outcomes;
(4) Identify key limitations, inconsistencies, and sources of bias in the current literature, including issues related to standardization, reproducibility, and safety;
(5) Highlight gaps in knowledge and define priorities for future research, particularly in relation to well-designed randomized controlled trials and long-term safety assessment.

Through this focused and critical approach, the review aims to clarify the current position of ozone therapy within evidence-based medicine and to distinguish between its experimental rationale and its clinically established role.

## 3 Materials and methods

This narrative review was conducted to provide a structured and critical synthesis of current evidence on the mechanisms of action, biochemical effects, and clinical applications of medical ozone therapy. A comprehensive literature search was performed using major electronic databases, including PubMed, Scopus, and Web of Science. The search strategy combined keywords and Medical Subject Headings (MeSH) related to ozone therapy and its biological and clinical effects, including “ozone therapy,” “oxygen–ozone,” “oxidative stress,” “redox signaling,” “inflammation,” “clinical trial,” and “systematic review.” The search was limited to articles published in English. Priority was given to studies published within the last two decades, although earlier publications were included when relevant to foundational mechanistic concepts. Studies were selected based on their relevance to the objectives of the review, focusing on three main domains: (1) mechanisms of action and biochemical interactions; (2) effects on blood biochemistry and cellular metabolism; (3) clinical applications across different medical fields.

To enhance the interpretability and reliability of the presented evidence, priority was given to higher levels of evidence, including randomized controlled trials (RCTs), systematic reviews, and meta-analyses. Observational studies, case series, and experimental research were also included to provide a comprehensive overview of emerging data and mechanistic insights, particularly in areas where clinical evidence remains limited. Although this is a narrative review and does not follow a formal systematic review protocol, efforts were made to apply a structured and transparent approach to literature selection and synthesis. A formal risk of bias assessment was not performed; however, methodological limitations of included studies such as small sample size, lack of control groups, heterogeneity of protocols, and potential sources of bias were critically considered during interpretation of the findings. Due to the heterogeneity of the available literature, no quantitative synthesis (meta-analysis) was performed. Instead, findings were qualitatively analyzed and integrated, with particular emphasis on distinguishing between experimental evidence and clinically validated outcomes. To improve clarity and facilitate comparison across clinical studies, representative studies were summarized in Table 1, including information on study design, sample size, main outcomes, and level of evidence. This approach highlights both the potential clinical applications and the limitations of the current evidence base.

**TABLE 1:** Representative clinical evidence on ozone therapy across selected indications, including study design, sample size, and level of evidence.

References	Indication	Study type	Sample size	Main outcomes	Level of evidence	Key limitations
Wen et al. (112)	Chronic wounds	Systematic review and meta-analysis	Multiple studies	Improved wound healing parameters	Moderate	High heterogeneity, small trials
Lino et al. (11)	Knee osteoarthritis	Umbrella review (RCTs)	15 RCTs	Mixed effects on pain and function	Moderate–high	Inconsistent endpoints, protocol variability
de Andrade et al. (114)	Low back pain	Systematic review and meta-analysis	Multiple RCTs	Modest pain reduction	Moderate	Risk of bias, short follow-up
Serra et al. (116)	Multiple indications	Evidence and gap map	26 reviews	Overall inconsistent evidence	Low–moderate	Variable study quality
Lima Bastos et al. (118)	Dentistry	Systematic review	Multiple studies	Antimicrobial and clinical effects	Low–moderate	Lack of high-quality trials
Alimohammadi et al. (24)	Inflammatory diseases	Meta-analysis (RCTs)	Multiple RCTs	Improved oxidative stress markers	Moderate	Limited clinical relevance
Di Paolo et al. (28)	Peripheral arterial disease	Controlled clinical study	40 patients	Improved walking distance	Low–moderate	Small sample, limited follow-up
Martínez-Sánchez et al. (107)	Diabetic foot ulcers	Controlled clinical study	100 patients	Enhanced healing, reduced infection	Moderate	Limited blinding
Hernández et al., (44)	COVID-19 pneumonia	Prospective cohort study	30 patients	Improved oxygenation markers	Low	No randomization
Andreula et al. (68)	Lumbar disk herniation	Prospective clinical study	∼100 patients	Pain and mobility improvement	Low–moderate	No control group
Borrelli et al. (5)	Macular degeneration	Randomized controlled trial	60 patients	Improved visual parameters	Moderate	Small sample size
Jeyaraman et al. (4)	Musculoskeletal disorders	Narrative/systematic review	Multiple studies	Reported symptomatic benefit	Low–moderate	Heterogeneity, lack of standardization

## 4 Mechanism of action and biochemical interactions of ozone

**FIGURE 1:**
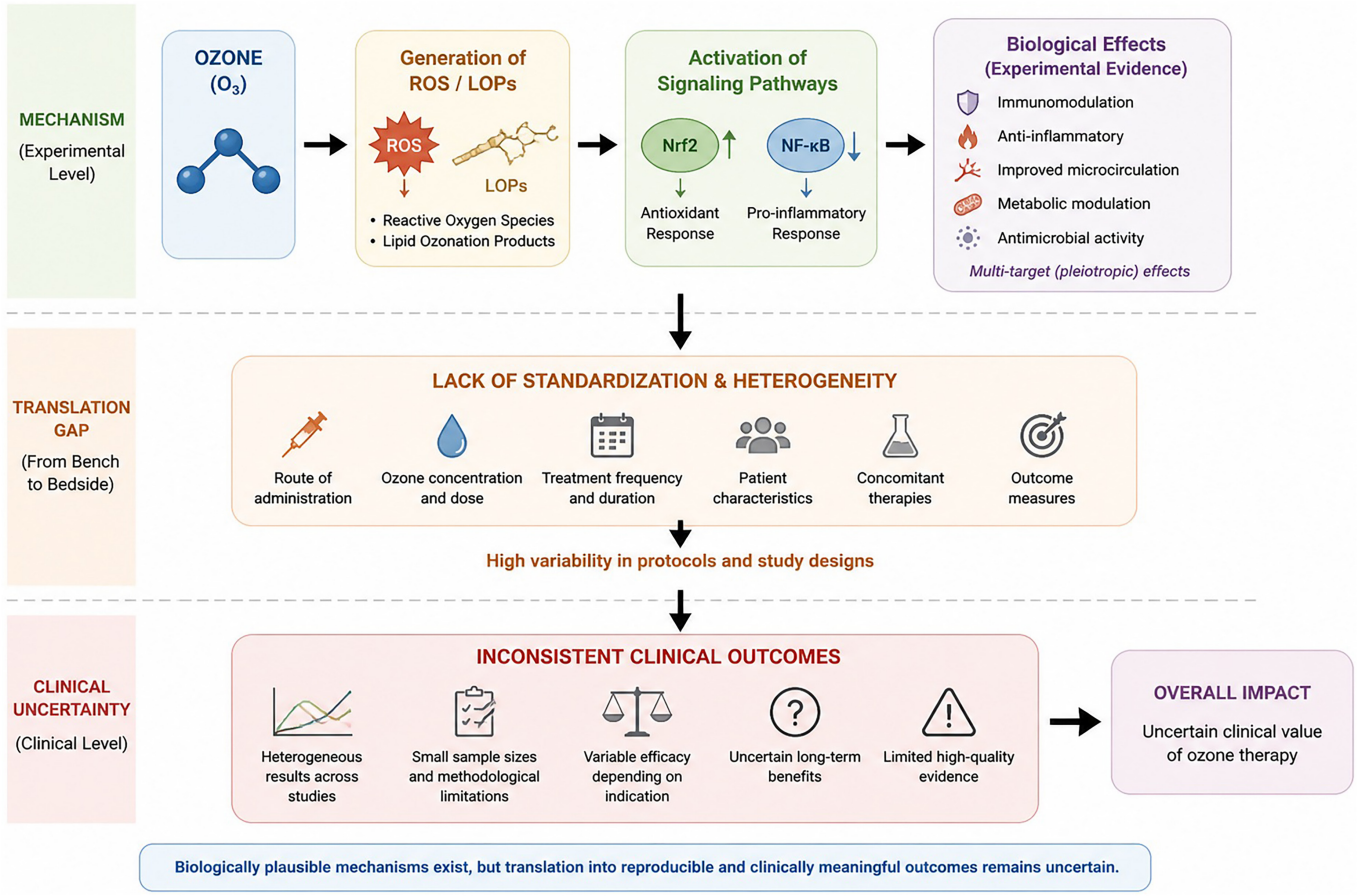
Mechanistic framework and translational gap in ozone therapy.

The biological effects of medical ozone are primarily attributed to its strong oxidative capacity and its rapid reactions with biomolecules in biological fluids. Due to its high reactivity, ozone does not persist in tissues but reacts immediately with lipids, proteins, and antioxidants, leading to the formation of reactive oxygen species (ROS), including hydrogen peroxide (H_2_O_2_), and lipid ozonation products (LOPs). These secondary products are considered key mediators of ozone-induced biological effects (17–19). At the experimental level, ROS and LOPs act as signaling molecules capable of modulating cellular pathways involved in oxidative stress responses, inflammation, and metabolic regulation. In particular, activation of redox-sensitive transcription factors such as nuclear factor erythroid 2–related factor 2 (Nrf2) and nuclear factor kappa B (NF-κB) has been widely described *in vitro* and in animal studies (6, 20–23). Through these pathways, ozone exposure has been associated with upregulation of antioxidant enzymes, modulation of cytokine production, and alterations in cellular redox balance (24, 25). However, it is important to emphasize that most of these mechanisms have been characterized under controlled experimental conditions, and their direct translation into clinically meaningful effects remains uncertain. Evidence synthesis studies indicate that, despite extensive mechanistic data, the link between redox modulation and clinical outcomes has not been clearly established (26, 27). Recent hypotheses suggest that ozone or ozone-derived species may exert more direct effects on cellular signaling than previously assumed. In particular, interactions with membrane-associated structures, including transient receptor potential (TRP) cation channels, have been proposed as potential mechanisms of cellular activation and signal transduction (28). While these concepts expand the current understanding of ozone biology, they remain largely theoretical and require further experimental and clinical validation. Recent evidence also suggests that ozone or ozone-like reactive species may be generated endogenously as part of physiological redox processes. This concept challenges the traditional view of ozone solely as an exogenous agent and introduces the possibility that ozone-related signaling may occur under physiological conditions. For example, endogenous ozone formation has been proposed as a component of reactive oxygen species (ROS) biology, potentially contributing to cellular signaling and oxidative regulation. However, this hypothesis remains under active investigation, and its relevance to therapeutic ozone administration is not yet clearly established. The extent to which exogenous medical ozone mimics or amplifies endogenous oxidative signaling pathways requires further experimental and clinical validation. The concept of ozone-induced “controlled oxidative stress” has been proposed to explain its potential therapeutic effects. According to this model, low to moderate oxidative stimuli may trigger adaptive responses that enhance cellular antioxidant capacity and resilience (29–31). However, this hormetic framework remains debated, and its clinical applicability is uncertain, particularly given the narrow therapeutic window of ozone and the risk of oxidative damage at higher concentrations. Furthermore, the biochemical interactions of ozone are influenced by the biological environment in which it is applied. Factors such as plasma composition, tissue antioxidant capacity, and lipid content may significantly affect the formation and activity of secondary mediators. This variability introduces additional uncertainty regarding the reproducibility and predictability of ozone-induced effects across different biological and clinical settings (32, 33). In summary, the mechanisms of action of ozone are relatively well-characterized at the experimental level and involve complex redox-dependent signaling processes mediated by ROS and LOPs. However, the clinical significance of these mechanisms remains incompletely understood. A clear distinction between experimentally observed effects and clinically validated outcomes is essential when interpreting the potential therapeutic role of medical ozone therapy. Schematic representation of the proposed biological effects of medical ozone and the discrepancy between experimental mechanisms and clinical outcomes Figure 1. Ozone exposure leads to the formation of reactive oxygen species (ROS) and lipid ozonation products (LOPs), which activate redox-sensitive signaling pathways, including Nrf2 and NF-κB, resulting in a range of biological effects observed under experimental conditions (6). However, the translation of these mechanisms into clinical practice is limited by a lack of standardization in treatment protocols, including variability in ozone concentration, route of administration, treatment frequency, and patient selection. This heterogeneity contributes to inconsistent clinical outcomes and uncertainty regarding therapeutic efficacy. The figure highlights the central challenge of medical ozone therapy: biologically plausible mechanisms that are not consistently translated into reproducible and clinically validated clinical benefits.

## 5 Ozone, blood biochemistry, and cellular metabolism

When medical ozone is introduced into the bloodstream, it reacts rapidly with plasma components, leading to the formation of reactive oxygen species (ROS), such as hydrogen peroxide (H_2_O_2_), and lipid ozonation products (LOPs). Due to its high reactivity, ozone itself does not persist in biological systems, and its biological effects are mediated primarily through these secondary products (34, 35). At the experimental level, these ROS and LOPs act as signaling molecules capable of influencing cellular redox balance and metabolic pathways. Erythrocytes have been proposed as key targets of ozone-induced biochemical modulation. *In vitro* and *ex vivo* studies suggest that controlled oxidative interactions with membrane lipids and proteins may affect erythrocyte deformability, membrane fluidity, and aggregation behavior, potentially influencing blood rheology and microcirculatory fl. However, it is important to emphasize that most of these findings are derived from experimental or *ex vivo* conditions, and their clinical relevance remains uncertain. Evidence from controlled clinical studies confirming consistent improvements in blood rheology or tissue perfusion following ozone therapy is limited. Ozone exposure has also been associated with shifts in the oxyhemoglobin dissociation curve, potentially facilitating oxygen release to peripheral tissues. In addition, experimental studies suggest activation of metabolic pathways within erythrocytes, including glycolysis and the pentose phosphate pathway, with increased production of 2,3-diphosphoglycerate (2,3-DPG) and nicotinamide adenine dinucleotide phosphate (NADPH) (36, 37). These mechanisms are hypothesized to support oxygen delivery and intracellular antioxidant capacity. Nevertheless, the extent to which these biochemical changes translate into clinically meaningful improvements in oxygen utilization remains unclear. Clinical data supporting enhanced tissue oxygenation or metabolic efficiency are limited and often derived from small or methodologically heterogeneous studies (38, 39). Beyond erythrocytes, ozone-derived mediators may influence cellular signaling in circulating and tissue cells through activation of redox-sensitive pathways, including Nrf2-dependent mechanisms. These pathways are associated with upregulation of antioxidant enzymes and modulation of oxidative stress responses (6, 40, 41). However, these effects have been primarily demonstrated in experimental models, and their reproducibility in clinical settings has not been consistently established. Importantly, the biochemical effects of ozone are highly dose-dependent. Low to moderate concentrations have been proposed to induce adaptive responses consistent with hormesis, whereas higher concentrations may result in oxidative damage and cellular dysfunction (42, 43). This narrow therapeutic window introduces variability and limits the predictability of treatment outcomes. In summary, ozone-induced biochemical interactions in blood are relatively well-characterized at the experimental level and involve complex redox-dependent signaling processes (14, 20, 21, 25–28). However, the clinical significance of these mechanisms remains uncertain. A cautious interpretation is required, particularly given the limited availability of high-quality clinical evidence and the variability of treatment protocols (44–47).

## 6 Pleiotropic effects of medical ozone

The concept of pleiotropy has been used to describe the wide range of biological effects attributed to medical ozone, including antimicrobial activity (encompassing antibacterial, antifungal, and antiviral effects), immunomodulation, anti-inflammatory responses, and effects on microcirculation. These effects are generally linked to the interaction of ozone-derived reactive oxygen species (ROS) and lipid ozonation products (LOPs) with multiple cellular pathways (48–51). At the experimental level, ozone-induced redox signaling has been associated with modulation of oxidative stress responses, activation of antioxidant defense systems, and changes in cytokine production (52, 53). However, most of these findings originate from *in vitro* or preclinical studies and may not reflect the complexity of human physiology. Emerging evidence suggests that ozone or ozone-related reactive species may also be generated endogenously as part of physiological redox processes, further complicating the interpretation of pleiotropic effects attributed to exogenous ozone therapy. The direction and magnitude of ozone-induced responses appear to depend on factors such as dose, route of administration, tissue environment, and baseline oxidative status, limiting the predictability of outcomes (54, 55). Indeed, similar interventions have been associated with divergent biological effects, particularly in immune and inflammatory responses (56, 57). From a clinical perspective, the pleiotropic model may oversimplify the relationship between molecular effects and patient outcomes. While multi-target activity suggests potential versatility, it also introduces uncertainty regarding specificity and reproducibility. Clinical evidence remains limited and heterogeneous, with most studies characterized by small sample sizes and variable methodologies (58, 59). In summary, although pleiotropic effects of ozone are well-described at the experimental level, their clinical relevance remains uncertain. Variability, context dependency, and limited reproducibility warrant cautious interpretation and highlight the need for well-designed studies linking specific mechanisms to defined clinical outcomes.

## 7 Antimicrobial effects, including antiviral activity

Ozone has been widely investigated for its antimicrobial properties, primarily due to its strong oxidative capacity. Through reactions with microbial cell membranes, proteins, and nucleic acids, ozone can induce structural damage and functional inactivation of bacteria, fungi, and viruses. These effects have been consistently demonstrated under *in vitro* conditions and in controlled experimental environments (60, 61). The antimicrobial action of ozone is considered non-specific and involves oxidative disruption of cell walls, lipid peroxidation, and alteration of intracellular components. In the case of viruses, ozone has been proposed to affect viral capsid proteins and envelope lipids, potentially impairing viral entry and replication. These mechanisms provide a biologically plausible basis for antimicrobial activity (14, 37, 62, 63). However, it is important to emphasize that the majority of evidence supporting antimicrobial and antiviral effects of ozone is derived from experimental models. The conditions under which these effects are observed such as direct exposure, controlled concentrations, and absence of complex biological barriers differ substantially from clinical settings. Consequently, the magnitude of antimicrobial effects observed *in vitro* may not be directly reproducible *in vivo*. In clinical practice, multiple factors including limited tissue penetration, interaction with biological fluids, and host immune responses may significantly modify or reduce the effectiveness of ozone. As a result, the translation of laboratory findings into clinically meaningful antimicrobial outcomes remains uncertain (64, 65). Clinical evidence evaluating antimicrobial efficacy is limited and heterogeneous. Some studies suggest potential benefits in the management of infected wounds, periodontal disease, and localized infections; however, these findings are typically based on small clinical trials or observational studies with variable methodologies and limited standardization (66, 67). In the context of viral infections, including emerging diseases such as COVID-19, ozone therapy has been proposed as an adjunctive intervention based on its potential effects on oxidative stress and immune modulation. Nevertheless, available evidence is limited to small, uncontrolled studies, and no robust clinical trials have demonstrated a direct antiviral effect in humans (16, 43–48). Overall, while ozone demonstrates clear antimicrobial activity under experimental conditions, its clinical relevance remains uncertain. The current evidence does not support the use of ozone therapy as a primary antimicrobial or antiviral treatment. Instead, it may be considered an adjunctive approach in selected clinical contexts, pending further validation through well-designed clinical trials (68, 69).

## 8 Immunomodulatory effects and cytokine regulation

The immunomodulatory effects of ozone therapy have been proposed as one of its key biological properties, primarily based on its influence on redox-sensitive signaling pathways. Experimental studies suggest that ozone-derived reactive oxygen species (ROS) and lipid ozonation products (LOPs) may modulate immune cell function and cytokine production through activation of transcription factors such as nuclear factor erythroid 2–related factor 2 (Nrf2) and nuclear factor kappa B (NF-κB) (6, 70–73). At the experimental level, ozone exposure has been associated with changes in the production of both pro-inflammatory and anti-inflammatory cytokines, including interleukins (e.g., IL-6, IL-8, IL-10) and tumor necrosis factor alpha (TNF-α). These findings have led to the hypothesis that ozone may influence the balance between inflammatory and anti-inflammatory signaling pathways (6, 74–82). Emerging evidence also suggests that ozone or ozone-related reactive species may be generated endogenously and participate in physiological redox signaling. This concept introduces additional complexity in interpreting the effects of exogenous ozone therapy, as it raises the possibility that some observed biological responses may overlap with intrinsic oxidative signaling mechanisms. However, the interpretation of these effects is complicated by their bidirectional nature. Depending on experimental conditions, ozone has been reported to induce both pro-inflammatory and anti-inflammatory responses. Factors such as dose, duration of exposure, cellular context, and baseline inflammatory status appear to play a critical role in determining the direction of the response. This variability limits the predictability of immunological outcomes. Importantly, most evidence supporting immunomodulatory effects is derived from *in vitro* studies or animal models. The extent to which these findings translate into clinically meaningful immune modulation in humans remains unclear. Evidence synthesis studies indicate that reported immunological effects are inconsistent and often based on small or methodologically heterogeneous studies (16, 83–90). Moreover, the clinical relevance of cytokine modulation is highly context-dependent and varies across disease states. While modulation of inflammatory pathways may be beneficial in certain chronic inflammatory conditions, inappropriate or poorly controlled immune activation could be detrimental in others. The current literature does not provide sufficient evidence to define clear, disease-specific immunological effects of ozone therapy. In addition, methodological heterogeneity across studies including differences in ozone concentration, routes of administration, and treatment protocols further complicates interpretation of immunological findings. This lack of standardization limits reproducibility and makes comparison between studies difficult. In summary, ozone therapy has been shown to influence immune-related pathways and cytokine production under experimental conditions. However, the clinical significance of these effects remains uncertain due to variability, context dependency, and limited high-quality clinical evidence. A cautious and disease-specific approach is required when interpreting the potential immunomodulatory role of ozone therapy.

## 9 Anti-inflammatory and analgesic effects

The potential anti-inflammatory and analgesic effects of ozone therapy have been attributed to its influence on redox-sensitive signaling pathways and the modulation of inflammatory mediators. Experimental studies indicate that ozone-derived reactive oxygen species (ROS) and lipid ozonation products (LOPs) may affect cytokine production, oxidative stress responses, and nociceptive signaling pathways (91–94). At the preclinical level, ozone exposure has been associated with reduced expression of pro-inflammatory mediators and modulation of pain-related pathways. These findings provide a mechanistic rationale for potential therapeutic effects; however, they have been predominantly observed under controlled experimental conditions and their clinical translation remains uncertain (95, 96). Clinical evidence evaluating these effects is primarily derived from studies in musculoskeletal conditions, particularly osteoarthritis and low back pain. Randomized controlled trials (RCTs), meta-analyses, and observational studies have reported reductions in pain intensity and improvements in functional outcomes following local ozone administration (96–98). Nevertheless, these findings are inconsistent and should be interpreted with caution. Importantly, direct comparisons between ozone therapy and established treatments such as non-steroidal anti-inflammatory drugs (NSAIDs), corticosteroid injections, or physical therapy are limited. Where such comparisons have been conducted, no clear superiority of ozone therapy has been demonstrated (99–112). Furthermore, substantial heterogeneity exists across studies in terms of ozone concentration, route of administration, treatment frequency, and patient selection. These data highlight the variability in study design and outcomes, as well as the overall limitations of the current evidence base. Overall, while ozone therapy demonstrates biologically plausible mechanisms for anti-inflammatory and analgesic effects, the clinical evidence remains limited and heterogeneous. High-quality randomized controlled trials are scarce, and the available data do not support definitive conclusions regarding its efficacy. Therefore, ozone therapy should be considered a potential adjunctive approach rather than a replacement for established pain management strategies.

## 10 Modes of medical ozone administration

Ozone therapy can be administered through various systemic and local routes, depending on the intended clinical application. These include systemic approaches such as major autohemotherapy and rectal insufflation, as well as local techniques involving injections or topical applications. The selection of administration route is generally guided by the targeted tissue, proposed mechanism of action, and practical considerations. However, despite the wide range of described administration methods, there is a lack of standardized, evidence-based criteria for selecting specific approaches. Most administration strategies have been developed empirically and are supported by heterogeneous data derived from experimental studies, small clinical trials, or observational reports (3, 5, 7, 23, 31). Moreover, the relationship between route of administration, systemic bioavailability of ozone-derived mediators, and clinical outcomes remains incompletely understood. Differences in absorption, distribution, and local versus systemic effects may significantly influence therapeutic responses, but these aspects have not been consistently evaluated in controlled studies. In summary, while multiple modes of ozone administration are described in the literature and clinical practice, their comparative effectiveness and optimal clinical use remain uncertain. The absence of standardized protocols and high-quality comparative studies represents a major limitation in interpreting the clinical relevance of different administration routes (113–115).

### 10.1 Systemic administration

Systemic administration of ozone therapy is most commonly performed using major autohemotherapy (MAH), which involves the withdrawal of a defined volume of venous blood, exposure to a controlled oxygen–ozone gas mixture, and reinfusion into the patient. This approach is intended to facilitate systemic distribution of ozone-derived reactive mediators rather than direct exposure of tissues to ozone itself (1, 16, 113). Alternative systemic methods include rectal insufflation, which has been proposed as a less invasive option, particularly in situations where venous access is limited or contraindicated. However, the extent of systemic absorption, bioavailability, and equivalence to autohemotherapy remain incompletely understood and have not been consistently established in clinical studies (17, 113). Minor autohemotherapy, involving reinjection of a small volume of ozonated blood, has also been described, primarily in the context of immunomodulation. Nevertheless, its clinical role remains poorly defined due to the limited availability of controlled studies (9, 113). Although systemic ozone administration is widely used in clinical practice, the supporting evidence is heterogeneous and often based on small trials, observational studies, or indirect mechanistic data. The relationship between administered dose, biological effects, and clinical outcomes remains insufficiently characterized. In summary, systemic administration represents a central component of ozone therapy practice; however, its clinical effectiveness, optimal dosing strategies, and comparative efficacy between different administration routes remain uncertain. Further well-designed randomized controlled trials are required to clarify its therapeutic role and to support evidence-based recommendations.

### 10.2 Local administration

Local administration of ozone therapy is applied in a range of clinical contexts, including musculoskeletal disorders, pain management, dentistry, and wound care. Common techniques include intra-articular, periarticular, subcutaneous, and intramuscular injections, as well as topical application in the form of ozonated oils, ozonated water, or gaseous ozone delivered in closed systems (5, 25, 113). In musculoskeletal conditions, intra-articular and periarticular injections are among the most frequently described approaches, particularly in the management of low back pain and osteoarthritis. While some randomized and controlled studies suggest potential symptomatic benefits, the overall quality of evidence remains heterogeneous, with variability in study design, dosing protocols, and outcome measures (114–118). Topical ozone applications are widely used in dermatology, wound care, and dentistry, where they are proposed to combine antimicrobial effects with potential stimulation of tissue repair. However, much of the supporting evidence is derived from *in vitro* studies, small clinical trials, or observational data, limiting the strength of clinical recommendations (89, 113, 119). The selection of administration route, dosing parameters, and treatment frequency is not standardized and is often based on empirical clinical practice rather than validated protocols. This variability complicates comparison across studies and limits reproducibility of reported outcomes. In summary, local ozone administration is widely used and supported by a growing body of experimental and clinical data; however, the evidence base remains inconsistent. Further well-designed, adequately powered randomized controlled trials are needed to establish clear indications, optimize treatment parameters, and confirm clinical effectiveness.

### 10.3 Topical administration

Topical ozone application involves the use of ozonated water, ozonated oils, or controlled gas exposure, primarily in dermatological and wound care settings. These methods are intended to utilize the antimicrobial and local tissue-modulating properties of ozone. Nevertheless, clinical evidence supporting topical applications is limited and largely based on small or observational studies (26, 27, 113–115). Additionally, the relationship between the route of administration and clinical outcomes remains unclear. Biological effects may vary depending on concentration, exposure time, and the local tissue environment, and these variables are inconsistently reported. Across all administration routes, a major limitation is the lack of standardized dosing regimens and treatment protocols. Variability in ozone concentration, frequency of application, and duration of treatment contributes to substantial heterogeneity in the literature, making direct comparisons between studies difficult. Regulatory approaches to ozone administration also differ significantly between countries, reflecting ongoing uncertainty regarding its safety and clinical value. This variability further limits generalizability and highlights the absence of a unified clinical framework. In summary, multiple routes of ozone administration have been described, each with distinct theoretical and practical considerations. However, the absence of standardization, limited comparative evidence, and methodological heterogeneity prevent clear conclusions regarding optimal administration strategies. These approaches should therefore be interpreted within an exploratory and investigational context.

## 11 Therapeutic protocols

The therapeutic efficacy and safety of medical ozone therapy are considered to depend on the use of well-defined and standardized protocols. However, in practice, considerable variability exists in ozone concentration, route of administration, treatment frequency, and overall duration. This heterogeneity has historically limited the comparability of clinical studies and the reproducibility of reported outcomes (113–115). Although protocol-based approaches are commonly described in the literature and clinical practice, most are derived from empirical experience rather than high-quality evidence. As a result, many currently used regimens lack robust validation in randomized controlled trials. Consequently, while treatment protocols are often tailored to specific indications and individual patient characteristics, the absence of universally accepted standards introduces uncertainty regarding optimal dosing strategies and clinical effectiveness. This variability represents a major limitation in the interpretation of clinical outcomes and highlights the need for further standardization and evidence-based guidance.

### 11.1 General principles

Medical ozone is generated from pure oxygen using certified medical devices, and its concentration is expressed in micrograms per milliliter (μg/mL) within an oxygen–ozone gas mixture. Due to its high reactivity, ozone does not persist in biological systems, and its biological effects are mediated primarily through secondary reactive products. Ozone exhibits a dose-dependent effect that is often described within a hormetic framework, whereby low to moderate concentrations are proposed to induce adaptive biological responses, while higher concentrations may result in oxidative damage and cytotoxicity (2, 4, 16, 47). However, the clinical relevance and reproducibility of this hormetic model remain incompletely established, as most supporting evidence is derived from experimental and preclinical studies. The concept of a “therapeutic window” is frequently emphasized in ozone therapy, but its precise definition is not standardized and may vary depending on the route of administration, tissue environment, and patient-specific factors. This variability introduces uncertainty in dose selection and limits the predictability of clinical outcomes. Consequently, precise dosing and controlled administration are considered essential for minimizing potential risks. Nevertheless, the lack of universally accepted dosing standards and validated protocols remains a significant limitation in both clinical practice and research settings.

### 11.2 Systemic protocols

Systemic administration of ozone therapy most commonly includes major autohemotherapy (MAH), which involves the withdrawal of a defined volume of venous blood, exposure to a controlled oxygen–ozone gas mixture, and reinfusion into the patient. In clinical practice, treatment schedules are typically described as 1–3 sessions per week over several weeks; however, these regimens are largely based on empirical protocols rather than high-quality evidence (1, 11, 113–115). Rectal insufflation has been proposed as a less invasive alternative to systemic administration. Reported protocols commonly include ozone concentrations in the range of 10–30 μg/mL and variable gas volumes per session. This approach is often considered in situations where venous access is limited or contraindicated, although its systemic bioavailability and clinical equivalence to autohemotherapy remain insufficiently established (17, 113). Minor autohemotherapy, which involves reinjection of a small volume of ozonated blood, has been described primarily in the context of immunomodulation. However, supporting clinical evidence for this approach is limited, and its therapeutic role has not been clearly defined in controlled studies (9, 113). Across systemic protocols, substantial heterogeneity exists in terms of dosing strategies, treatment frequency, and duration. This variability reflects the lack of standardized, evidence-based guidelines and complicates comparison across clinical studies. Moreover, the relationship between administered ozone dose and clinical outcomes remains incompletely understood. In summary, while systemic ozone administration is widely practiced, current protocols are largely derived from clinical experience and observational data rather than robust randomized trials. Further well-designed studies are required to establish optimal dosing strategies, clarify mechanisms of action *in vivo*, and determine the clinical effectiveness of different systemic approaches.

### 11.3 Local and topical protocols

Local administration of ozone is commonly used in musculoskeletal disorders, pain management, dentistry, and wound care. Techniques include intra-articular, periarticular, subcutaneous, and intramuscular injections, as well as topical applications such as ozonated oils, ozonated water, and gaseous ozone exposure in closed systems (5, 25, 113). In musculoskeletal conditions, intra-articular and periarticular injections are frequently applied using low to moderate ozone concentrations, with volumes adjusted according to joint size and anatomical considerations. Subcutaneous and intramuscular techniques are also used in pain-related conditions. However, the selection of dosing parameters and treatment regimens is largely based on empirical practice and varies substantially across studies and clinical settings (113–115). Topical ozone therapy is widely employed in wound management, dermatology, and dentistry, where it is proposed to combine antimicrobial effects with potential stimulation of tissue repair processes (89, 103, 119). Ozonated oils and water are commonly used for their ease of application and presumed safety profile, although their composition and stability may vary depending on preparation methods. Despite widespread clinical use, the evidence supporting local and topical ozone applications remains heterogeneous. Some randomized and controlled studies particularly in musculoskeletal conditions such as low back pain or knee osteoarthritis suggest potential benefits; however, these findings are often limited by methodological variability, small sample sizes, and inconsistent outcome measures (114, 115). In dentistry and dermatology, most available data are derived from *in vitro* studies, small clinical trials, or observational reports, which limits the strength of clinical recommendations (89, 119). Similarly, in wound care, although ozone has demonstrated antimicrobial and healing-related effects under experimental conditions, the translation into consistent clinical benefit remains uncertain (113). In summary, local and topical ozone protocols are widely used across multiple clinical fields, but their application is characterized by significant variability and limited standardization. While some clinical studies suggest potential benefits, the overall quality of evidence remains moderate to low. Further well-designed, adequately powered randomized trials are needed to establish optimal treatment parameters and confirm clinical efficacy.

### 11.4 Treatment duration and monitoring

Treatment protocols in ozone therapy typically consist of a series of sessions administered over several weeks, most commonly ranging from 10 to 15 applications, followed by clinical reassessment. In chronic conditions, maintenance therapy is sometimes proposed; however, optimal treatment duration and scheduling remain insufficiently defined and are largely based on empirical practice rather than robust clinical evidence (11, 113–115). The frequency and total number of sessions vary considerably depending on the indication, route of administration, and individual patient response. This variability reflects the lack of standardized, evidence-based protocols and contributes to heterogeneity across clinical studies. Monitoring during ozone therapy is not uniformly standardized. Clinical evaluation remains the primary method of assessing treatment response, while the use of laboratory markers such as indicators of oxidative stress, antioxidant capacity, or inflammatory status has been proposed in experimental and exploratory settings (26, 47). However, the clinical utility and predictive value of these biomarkers have not been consistently validated. Importantly, given the dose-dependent and potentially hormetic effects of ozone, careful monitoring is required to balance potential therapeutic benefits against the risk of oxidative stress and adverse effects. In practice, treatment adjustments are often individualized, but this approach is not supported by clearly defined criteria or validated monitoring frameworks. In summary, treatment duration and monitoring strategies in ozone therapy remain insufficiently standardized and are largely guided by clinical experience. The absence of validated protocols and objective monitoring parameters limits reproducibility and complicates the interpretation of clinical outcomes. Further research is needed to establish evidence-based recommendations for treatment scheduling and monitoring strategies.

### 11.5 Safety considerations

The safe application of ozone therapy requires strict adherence to standardized protocols, the use of certified medical ozone generators, and appropriate ozone-resistant materials. Due to its strong oxidative properties, ozone can exert both therapeutic and toxic effects depending on dose, route of administration, and exposure conditions (2, 4, 16, 47). Direct inhalation of ozone is contraindicated because of its well-documented pulmonary toxicity, including airway irritation, inflammation, and potential long-term respiratory damage (28, 120). Consequently, all medical applications must ensure controlled delivery systems that prevent accidental inhalation. The safety profile of ozone therapy is often described as favorable when appropriate protocols are followed; however, this assessment is largely based on observational data and clinical experience rather than high-quality randomized trials (113–115). Reported adverse effects are generally mild and transient, including local discomfort at the injection site, headache, fatigue, and, less frequently, vasovagal reactions (5, 6). Importantly, ozone exhibits a narrow therapeutic window, and inappropriate dosing may result in oxidative damage, lipid peroxidation, and cellular injury (16, 47). The dose-dependent nature of its biological effects introduces variability in both efficacy and safety, particularly in the absence of standardized treatment protocols. Furthermore, long-term safety data remain limited. There is insufficient evidence regarding cumulative oxidative effects, potential interactions with comorbid conditions, and the impact of repeated systemic administration over extended periods. Regulatory agencies, including the U.S. Food and Drug Administration (FDA), have not approved ozone therapy for medical use, citing insufficient evidence of safety and efficacy. In summary, while ozone therapy appears to be relatively safe when applied within controlled and protocol-based settings, its safety profile remains incompletely characterized. Careful patient selection, strict adherence to dosing protocols, and avoidance of inappropriate administration routes are essential. Further high-quality clinical studies are required to establish both short- and long-term safety and to better define risk benefit profiles across different clinical indications.

### 11.6 Clinical variability and limitations

Despite the availability of guideline recommendations, including those from organizations such as the Italian Scientific Society of Oxygen Ozone Therapy (SIOOT), substantial heterogeneity persists in clinical practice (11). Existing recommendations are largely based on expert consensus and practice-oriented frameworks rather than robust, high-quality clinical evidence. Significant variability exists in dosing strategies, ozone concentrations, routes of administration, treatment frequency, and duration of therapy. This lack of standardization limits comparability across studies, reduces reproducibility of results, and complicates the interpretation of clinical outcomes. Moreover, inconsistent reporting of treatment parameters in the literature further contributes to methodological uncertainty. Importantly, many proposed therapeutic protocols are derived from empirical clinical experience or extrapolation of experimental findings rather than from well-designed randomized controlled trials. As a result, the evidence supporting specific dosing regimens or treatment schedules remains insufficient. This variability also raises concerns regarding both efficacy and safety, as the therapeutic window of ozone is narrow and dose-dependent. In the absence of clearly defined and validated protocols, the predictability of clinical responses remains limited. In summary, while existing recommendations provide a practical framework for clinical application, ozone therapy protocols remain insufficiently standardized and not adequately supported by high-quality evidence. Further well-designed, adequately powered clinical studies with standardized methodologies are essential to establish optimal treatment parameters and to support the integration of ozone therapy into evidence-based clinical practice.

## 12 Clinical applications of medical ozone

The potential clinical applications of medical ozone therapy reflect its proposed biological effects, including antimicrobial activity, modulation of inflammation, and effects on microcirculation and tissue oxygenation. These effects are primarily attributed to redox-mediated mechanisms involving reactive oxygen species (ROS) and lipid ozonation products (LOPs), which have been extensively described in experimental studies. Representative clinical studies across different indications are summarized in Table 1 (4, 24, 28, 41, 55, 68, 107, 111–113, 115, 117).

Despite these mechanistic insights, the strength of clinical evidence varies substantially across different indications. Most available data are derived from small clinical studies, observational reports, or experimental research, while high-quality randomized controlled trials (RCTs) remain limited. Evidence synthesis studies, including systematic reviews and meta-analyses, consistently highlight substantial heterogeneity, methodological limitations, and variable quality of available data (26, 113–117). Importantly, the translation of experimental findings into clinically meaningful outcomes remains uncertain. Reported therapeutic effects are often context-dependent and influenced by factors such as dosing, route of administration, and patient-specific characteristics. This variability, combined with the lack of standardized treatment protocols, limits reproducibility and complicates interpretation of results across studies. Furthermore, although ozone therapy is used in a range of clinical settings, its role is most frequently described as adjunctive rather than primary. In some practice-oriented and regional contexts, it has been proposed as a standalone intervention; however, such approaches are not supported by robust clinical evidence and remain controversial (121–124). Overall, while ozone therapy demonstrates biologically plausible effects and potential clinical utility in selected conditions, the current evidence base remains insufficient to support its routine use across indications. A critical, evidence-based approach is required, with particular emphasis on distinguishing between experimental observations and clinically validated outcomes (125–130).

### 12.1 Infectious and wound-related conditions

Ozone therapy has been extensively investigated in infectious and wound-related conditions, primarily due to its strong oxidative and antimicrobial properties. Experimental studies demonstrate that ozone can inactivate a broad spectrum of microorganisms, including bacteria, fungi, and viruses, through oxidative damage to cell membranes, proteins, and nucleic acids (15, 62, 63, 128–131). In addition to its antimicrobial effects, ozone has been proposed to promote wound healing by modulating local inflammatory responses, improving tissue oxygenation, and stimulating cellular repair mechanisms (47, 132, 133). In clinical settings, ozone therapy has been applied in the management of chronic wounds, diabetic ulcers, infected lesions, and postoperative infections. Some clinical studies and observational reports suggest that ozone therapy may accelerate wound healing, reduce microbial burden, and improve local tissue conditions, particularly when used as an adjunct to standard wound care (81–83, 112, 131–135). These effects are often attributed to a combination of antimicrobial activity and modulation of oxidative stress at the tissue level. However, the clinical evidence supporting these applications remains heterogeneous and limited in quality. Many studies are characterized by small sample sizes, lack of appropriate control groups, and variability in treatment protocols, including differences in ozone concentration, delivery method, and duration of therapy. Systematic reviews evaluating ozone therapy in chronic wounds indicate potential benefits, but emphasize the need for cautious interpretation due to methodological limitations and risk of bias (112, 113). Importantly, the effectiveness of ozone therapy in infectious conditions is influenced by the complexity of the *in vivo* environment. Factors such as tissue penetration, interaction with biological fluids, and host immune responses may significantly modify its antimicrobial efficacy compared to controlled experimental conditions. Safety considerations in wound-related applications are generally favorable when ozone is applied topically or in controlled local settings. Nevertheless, improper dosing or excessive exposure may lead to oxidative tissue damage or delayed healing. Additionally, long-term outcomes and recurrence rates following ozone-based interventions remain insufficiently studied. Overall, while ozone therapy demonstrates clear antimicrobial and wound-healing effects under experimental conditions and may provide adjunctive benefits in selected clinical scenarios, current evidence does not support its use as a standalone treatment. Further well-designed randomized controlled trials with standardized protocols are required to establish its clinical efficacy and optimal application strategies.

### 12.2 Vascular and metabolic conditions

Ozone therapy has been investigated in vascular and metabolic disorders based on its proposed effects on blood rheology, oxygen delivery, and redox-mediated metabolic regulation. Experimental studies suggest that ozone-derived reactive oxygen species (ROS) and lipid ozonation products (LOPs) may influence endothelial function, erythrocyte deformability, and microcirculatory flow, as well as activate antioxidant defense pathways (21–23, 27, 29, 47). In peripheral vascular diseases, including chronic limb ischemia and intermittent claudication, ozone therapy has been explored as an adjunctive intervention. Some clinical studies and controlled trials report improvements in symptoms, walking distance, and peripheral perfusion following systemic ozone administration, particularly via autohemotherapy (30, 31, 51, 126). However, these studies are generally small and heterogeneous, limiting the strength and generalizability of conclusions. In metabolic disorders, particularly diabetes mellitus and its complications, ozone therapy has been investigated for its potential effects on oxidative stress, inflammation, and tissue oxygenation. Clinical studies in patients with diabetic foot and chronic ulcerations suggest possible benefits in wound healing and reduction of oxidative stress markers (81–83, 136). Additionally, meta-analyses and systematic reviews indicate that ozone therapy may influence oxidative stress indices in chronic inflammatory and metabolic conditions; however, the clinical significance of these effects remains uncertain due to heterogeneity and variable study quality (26, 137). The proposed metabolic effects of ozone include activation of glycolytic pathways, modulation of redox balance, and enhancement of cellular antioxidant capacity. While these mechanisms are supported by experimental data, their translation into consistent clinical improvements in metabolic control or long-term outcomes has not been clearly established. Importantly, variability in treatment protocols including ozone concentration, route of administration, and treatment duration represents a major limitation across studies. This heterogeneity complicates direct comparison of results and limits reproducibility. Safety considerations are particularly relevant in patients with vascular and metabolic disorders, who often present with comorbidities and altered oxidative balance. While ozone therapy is generally described as well-tolerated in controlled settings, the potential for oxidative damage and endothelial dysfunction at higher doses remains a concern (121). Overall, ozone therapy may offer potential adjunctive benefits in selected vascular and metabolic conditions, particularly in relation to microcirculation and wound healing. However, current evidence remains limited and heterogeneous, and does not support its use as a primary therapeutic approach. Further high-quality, standardized clinical trials are required to clarify its role in this field.

### 12.3 Dentistry and dermatology

Medical ozone therapy has been widely explored in dentistry and dermatology, primarily due to its antimicrobial properties, including activity against bacteria, fungi, and viruses, as well as its potential effects on tissue repair. Its proposed mechanisms include oxidative disruption of microbial cell structures, modulation of local inflammatory responses, and stimulation of wound healing processes. These effects have been consistently demonstrated in experimental settings, particularly in relation to bacteria, fungi, and biofilm-forming microorganisms (11, 15, 62, 63, 100, 138–140). In dentistry, ozone has been investigated for the management of dental caries, periodontal disease, endodontic infections, and oral mucosal conditions. *In vitro* and clinical studies suggest that ozone may reduce microbial load and influence lesion progression, particularly in early carious lesions and infected root canals (84–103, 141). However, systematic reviews indicate that the clinical effectiveness of ozone in caries management and periodontal therapy remains inconsistent and, in many cases, not superior to conventional treatments (89, 122). In dermatology, ozone therapy has been applied in conditions such as chronic wounds, diabetic ulcers, fungal infections, and inflammatory skin diseases. Topical ozone applications, including ozonated oils and ozonated water, have been associated with antimicrobial effects and potential enhancement of tissue regeneration (124, 125, 142, 143). Some clinical studies report improved wound healing outcomes, particularly in chronic and infected wounds; however, these findings are largely derived from small studies and observational data. The potential benefits of ozone in both fields are often attributed to its combined antimicrobial and pro-healing effects. Nevertheless, these outcomes are highly dependent on application method, concentration, and exposure time, which vary significantly across studies. This lack of standardization limits reproducibility and complicates comparison between clinical trials. Importantly, evidence from systematic reviews and evidence-mapping studies highlights substantial heterogeneity and variable quality of available data in both dentistry and dermatology (23, 119, 122). While ozone may provide adjunctive benefits in selected cases, its superiority over established treatment modalities has not been consistently demonstrated. Safety considerations are generally favorable in topical applications when appropriate protocols are followed. However, improper use or excessive exposure may result in local irritation or oxidative tissue damage. Furthermore, long-term safety data in dermatological and intraoral applications remain limited (104, 118). Overall, ozone therapy in dentistry and dermatology shows promising antimicrobial and supportive effects under specific conditions. However, current evidence supports its role primarily as an adjunctive intervention rather than a replacement for conventional therapies. Further well-designed, standardized clinical studies are required to establish its efficacy and optimal application parameters.

### 12.4 Neurological and autoimmune disorders

Ozone therapy has been explored in neurological and autoimmune disorders based on its proposed effects on redox homeostasis, immune modulation, and microcirculation. Experimental studies suggest that ozone-derived reactive oxygen species (ROS) and lipid ozonation products (LOPs) may influence neuroinflammatory pathways and cellular signaling mechanisms, including the activation of redox-sensitive transcription factors such as Nrf2 (6, 19, 47, 78, 144). These mechanisms have been proposed as potentially relevant in conditions characterized by chronic inflammation and oxidative imbalance. In neurological disorders, including multiple sclerosis and ischemic conditions, ozone therapy has been investigated as an adjunctive intervention. Some small clinical studies and observational reports suggest possible improvements in functional parameters, cerebral metabolic activity, and markers of oxidative stress following systemic ozone administration (78–80). However, these findings are derived from limited and methodologically heterogeneous studies, often lacking appropriate control groups and long-term follow-up. In autoimmune diseases, ozone therapy has been proposed to modulate immune responses by influencing cytokine production and redox balance. Preliminary clinical observations and case reports describe potential benefits in conditions such as systemic sclerosis and Sjögren’s syndrome (74–76). Nevertheless, these reports are based on low levels of evidence and are subject to significant bias, limiting their clinical interpretability. Importantly, the immunomodulatory effects of ozone are bidirectional and highly context-dependent. Depending on dose, timing, and baseline inflammatory status, ozone exposure may induce either pro-inflammatory or anti-inflammatory responses (6, 121, 145). This variability introduces uncertainty regarding its therapeutic application in diseases characterized by complex and dysregulated immune activity. There is currently a lack of high-quality randomized controlled trials evaluating ozone therapy in neurological and autoimmune disorders. Evidence synthesis studies indicate that the overall quality of clinical evidence remains low to moderate, with substantial heterogeneity across indications (113–115). Safety considerations are particularly relevant in this patient population. The potential for unintended immune activation or exacerbation of autoimmune processes cannot be excluded, especially in the absence of clearly defined dosing parameters and long-term safety data (121, 146, 147). Overall, while ozone therapy demonstrates biologically plausible mechanisms that may be relevant to neurological and autoimmune diseases, current clinical evidence remains limited and inconclusive. Its use should be considered investigational and, if applied, restricted to adjunctive settings within carefully controlled clinical frameworks.

### 12.5 Oncology and supportive care

The potential role of ozone therapy in oncology has been explored primarily in the context of supportive care rather than as a direct anticancer treatment. Proposed mechanisms include modulation of oxidative stress, improvement of tissue oxygenation, and possible effects on immune regulation. These hypotheses are largely based on experimental studies and theoretical models suggesting that controlled oxidative stimuli may influence tumor microenvironment and cellular redox balance. Preclinical studies have demonstrated that ozone exposure may affect tumor growth, cellular metabolism, and oxygenation under controlled conditions. However, these findings are inconsistent and highly dependent on experimental design, ozone concentration, and tumor model. Importantly, the translation of these effects into clinically meaningful oncological outcomes remains uncertain (53, 69–73). Clinical evidence supporting the use of ozone therapy in oncology is limited and heterogeneous. Small clinical studies and observational reports have investigated ozone therapy as an adjunct to conventional treatments such as chemotherapy and radiotherapy, with some suggesting potential benefits in symptom control, quality of life, and treatment tolerance (70, 71). Nevertheless, these studies are characterized by small sample sizes, lack of appropriate control groups, and significant methodological variability. There is currently no high-quality evidence demonstrating that ozone therapy has a direct antitumor effect or improves survival outcomes in cancer patients. Claims regarding its use as an alternative or standalone oncological treatment are not supported by robust clinical data and should be interpreted with caution. Such claims are primarily found in case reports or practice-oriented publications and do not meet the standards of evidence-based oncology. In supportive care settings, ozone therapy has been proposed to alleviate cancer-related symptoms such as fatigue, pain, and hypoxia, as well as to potentially improve tissue oxygenation. While some preliminary studies report improvements in selected parameters, these findings remain inconsistent and require confirmation in well-designed randomized controlled trials (124, 148). Importantly, the use of ozone therapy in oncology raises safety considerations. The interaction between oxidative therapies and tumor biology is complex, and the potential for both beneficial and harmful effects cannot be excluded. Concerns include the possibility of promoting oxidative damage in healthy tissues, altering tumor behavior, or interfering with standard oncological treatments. Overall, current evidence does not support the use of ozone therapy as a primary treatment modality in oncology. Its role, if any, appears to be limited to adjunctive supportive care, and even in this context, the evidence remains insufficient. Further high-quality clinical studies with standardized protocols are required to clarify its potential benefits and risks in oncological practice.

### 12.6 Viral infections, including COVID-19

Viral effects of ozone should be understood as part of its broader antimicrobial activity. The potential application of ozone therapy in viral infections has been proposed based on its oxidative properties and possible effects on immune modulation and redox signaling. Experimental studies suggest that ozone may disrupt viral structures, including envelope lipids and capsid proteins, and influence host antiviral responses through modulation of oxidative stress pathways and cytokine production. However, these mechanisms are primarily derived from *in vitro* models and may not be directly translatable to clinical settings. During the COVID-19 pandemic, ozone therapy was investigated as a potential adjunctive treatment aimed at improving oxygenation, modulating inflammatory responses, and reducing disease severity. Several small observational studies and pilot clinical trials reported improvements in selected clinical parameters, including oxygen saturation, inflammatory markers, and hospitalization outcomes following systemic ozone administration, particularly via major autohemotherapy (32–45, 48, 60). However, these studies were characterized by limited sample sizes, lack of randomization, and significant heterogeneity in treatment protocols. Systematic reviews and meta-analyses evaluating ozone therapy in COVID-19 and other viral infections have highlighted the low quality and inconsistency of available evidence. While some analyses suggest potential adjunctive benefits, the overall certainty of evidence remains low due to methodological limitations, risk of bias, and heterogeneity across studies (116, 120, 149). Importantly, no high-quality randomized controlled trials have demonstrated a direct antiviral effect of ozone therapy in humans. The complexity of viral pathophysiology, including host–virus interactions, immune response variability, and tissue-specific factors, further limits the extrapolation of experimental findings to clinical practice. In addition, the use of ozone therapy in viral infections raises safety considerations, particularly in patients with severe systemic inflammation or compromised respiratory function. The oxidative properties of ozone may contribute to both beneficial and detrimental effects depending on dose, timing, and patient-specific factors, further complicating its clinical application. Overall, while ozone therapy demonstrates antiviral activity under experimental conditions, its clinical relevance in viral infections, including COVID-19, remains uncertain. Current evidence does not support its use as a primary antiviral treatment. At present, ozone therapy should be considered an adjunctive and investigational approach, requiring further validation in well-designed randomized controlled trials.

### 12.7 Geriatrics and rehabilitation

Ozone therapy has been explored in geriatric and rehabilitation settings, particularly in conditions associated with chronic pain, reduced mobility, impaired microcirculation, and delayed tissue repair. Proposed mechanisms include modulation of oxidative stress, improvement of oxygen delivery, and potential effects on inflammatory and metabolic pathways. These mechanisms are primarily derived from experimental data and require cautious interpretation in clinical contexts. In musculoskeletal disorders, including osteoarthritis and chronic low back pain, ozone therapy has been investigated as an adjunctive intervention. Some clinical studies suggest potential benefits in pain reduction and functional improvement, particularly following intra-articular or paravertebral administration (48–50, 113–115). However, these findings are often based on small randomized trials or observational studies with heterogeneous methodologies, limiting the strength of conclusions. In elderly patients with chronic wounds or impaired healing capacity, topical and systemic ozone applications have been proposed to enhance tissue repair through antimicrobial effects and modulation of local oxidative balance. Observational studies and small clinical series report improved wound healing outcomes, particularly in diabetic foot and chronic ulcerations (81–83, 112, 131–134). Nevertheless, these results should be interpreted cautiously due to variability in treatment protocols and limited methodological rigor. Rehabilitation-oriented applications have also been described, including potential improvements in muscle oxygenation, fatigue reduction, and functional recovery. Preliminary studies suggest that ozone therapy may influence metabolic and circulatory parameters; however, these findings are based on limited clinical evidence and require further validation (48, 127). Importantly, the elderly population may be particularly susceptible to oxidative stress and variability in redox homeostasis, which raises additional concerns regarding the safety and predictability of ozone therapy in this group. Age-related comorbidities and polypharmacy further complicate clinical application and risk assessment. Overall, while ozone therapy shows potential as an adjunctive intervention in geriatric and rehabilitation settings, current evidence remains limited and heterogeneous. Its clinical role is not clearly defined, and the lack of high-quality randomized controlled trials and standardized treatment protocols precludes firm conclusions. At present, ozone therapy in this context should be considered supportive and investigational rather than a primary therapeutic strategy.

### 12.8 Critical care and severe conditions

The use of ozone therapy in critically ill patients and severe clinical conditions has been explored primarily in the context of refractory infections, severe inflammatory states, and impaired tissue oxygenation. Proposed mechanisms include modulation of oxidative stress, potential effects on microcirculation, and immunoregulatory activity mediated by reactive oxygen species (ROS) and lipid ozonation products (LOPs). However, these mechanisms are largely extrapolated from experimental and preclinical studies, and their relevance in critically ill patients remains uncertain. During the COVID-19 pandemic, ozone therapy was investigated as a potential adjunctive treatment in patients with severe pneumonia and systemic inflammatory response. Small prospective and observational studies suggested possible improvements in oxygenation parameters, inflammatory markers, and clinical outcomes following systemic ozone administration, particularly in the form of major autohemotherapy (36–41, 117, 149). However, these studies were characterized by limited sample sizes, lack of randomization, and significant methodological heterogeneity. As a result, the reliability and generalizability of these findings are limited. In other critical conditions, including severe infections, ischemic disorders, and non-healing wounds, ozone therapy has been described in case reports and small clinical series. Some studies report potential benefits in tissue oxygenation and wound healing; however, these observations are based on low levels of evidence and are subject to substantial bias (30, 51, 108, 112, 131). Importantly, there is a lack of well-designed randomized controlled trials evaluating ozone therapy in intensive care settings. The application of ozone therapy in critically ill patients raises additional safety concerns. The physiological instability of these patients, combined with the oxidative properties of ozone, introduces potential risks related to redox imbalance, endothelial dysfunction, and unintended modulation of immune responses. Moreover, the absence of standardized protocols further complicates its use in acute and high-risk clinical scenarios. Overall, the current evidence does not support the routine use of ozone therapy in critical care or severe clinical conditions. While preliminary findings suggest potential adjunctive benefits, the lack of robust clinical trials, high-quality evidence, and standardized treatment protocols limits its clinical applicability. At present, ozone therapy in this context should be considered experimental and restricted to controlled clinical research settings.

### 12.9 Overall perspective

Across clinical applications, a consistent pattern emerges: biologically plausible mechanisms supported by experimental data, but limited, heterogeneous, and often inconsistent clinical evidence. It should be noted that evidence-based medicine encompasses not only randomized controlled trials but also observational studies and clinical expertise, which together contribute to the overall assessment of therapeutic interventions. Ozone therapy has a long history of clinical use supported by experimental research and observational data; however, the heterogeneity of these studies limits definitive conclusions regarding efficacy. These findings suggest that experimental and clinical observations provide a complementary, although not yet fully integrated, evidence base. Variability in study design, small sample sizes, and the lack of standardized protocols significantly restrict the strength of conclusions and limit comparability across studies. In most contemporary scientific literature, ozone therapy is described as an adjunctive approach rather than a substitute for established treatments (125–132). However, in certain clinical and regional contexts, particularly in settings where ozone therapy is more widely practiced and regulated, it has been proposed as a standalone therapeutic option. Such perspectives are reflected in selected clinical reports and practice-oriented publications within the field (139, 140, 142–145). Nevertheless, these approaches are generally based on observational studies, case series, or expert opinion and are not supported by high-quality randomized controlled trials or robust comparative evidence (26, 113–115, 127–132, 139, 140, 142–148). Additional uncertainty arises from the discrepancy between extensive mechanistic research and limited clinical validation. While redox-based biological effects have been consistently demonstrated under experimental conditions, their translation into reproducible therapeutic outcomes remains unclear. This translational gap has been highlighted in both experimental and clinical literature, particularly in studies addressing oxidative signaling, immune modulation, and metabolic effects (29–31, 121, 149, 150). Consequently, ozone therapy should be interpreted within an investigational framework, with its role primarily considered adjunctive. Although potential benefits have been reported in selected conditions, the current evidence base remains insufficient to support its routine use as a standalone therapeutic intervention. Further well-designed, adequately powered randomized controlled trials with standardized protocols are required to clarify its clinical role and determine whether specific indications may justify broader therapeutic application (26, 113–140, 142–149).

## 13 Safety considerations and potential risks

The safety profile of medical ozone therapy remains a subject of ongoing debate and is closely related to its strong oxidative properties. While controlled administration at low concentrations has been proposed to induce adaptive biological responses, ozone is a recognized toxic agent, particularly when inhaled, and its potential to cause oxidative damage must be carefully considered (29, 55, 125). One of the most well-established risks is associated with inhalation, which is contraindicated due to its irritative and toxic effects on the respiratory tract. Exposure to ozone gas can lead to airway inflammation, impaired pulmonary function, and oxidative injury to lung tissue. These effects are well-documented in environmental and occupational health studies and highlight the potential hazards of uncontrolled exposure (150). Even when administered via non-inhalational routes, ozone may induce oxidative stress if dosing is not carefully controlled. The concept of a therapeutic window is frequently cited; however, this window has not been clearly defined in clinical practice. Variability in ozone concentration, exposure time, and administration techniques introduces uncertainty and may increase the risk of adverse effects (42, 43). Reported adverse events associated with ozone therapy are generally described as mild and transient, including local discomfort, irritation, or headache. However, these observations are largely derived from small clinical studies or observational reports, which may underestimate the true incidence of complications due to limited follow-up and potential reporting bias (6, 11, 127, 128). Importantly, data on long-term safety are lacking. There is insufficient evidence regarding the cumulative effects of repeated ozone exposure, particularly in systemic applications. Potential concerns include chronic oxidative stress, endothelial dysfunction, and unintended modulation of immune responses. Experimental and translational studies suggest that prolonged oxidative imbalance may have deleterious biological consequences, although this has not been adequately evaluated in clinical settings (121, 149, 150). Furthermore, the lack of standardized treatment protocols contributes to variability in safety outcomes. Differences in dosing regimens, routes of administration, and clinical indications complicate the assessment of risk and make it difficult to establish clear safety recommendations (113–115, 134–140, 142–148). Regulatory perspectives on ozone therapy reflect these uncertainties. In several countries, including the United States, ozone therapy is not approved for medical use due to insufficient evidence of safety and efficacy, whereas in other regions it is practiced under varying degrees of regulation. This inconsistency underscores the absence of a unified consensus regarding its safety profile (15, 16, 127, 129). In summary, although ozone therapy is often described as safe when appropriately administered, this assertion is not supported by robust, high-quality evidence. The potential for oxidative toxicity, lack of long-term safety data, and absence of standardized protocols warrant a cautious and critical interpretation. At present, ozone therapy should be considered an investigational intervention with an uncertain risk benefit profile.

## 14 Summary

Medical ozone is associated with a wide range of proposed biological effects, primarily mediated by reactive oxygen species (ROS) and lipid ozonation products (LOPs) generated during its interaction with biological fluids. Experimental studies suggest that these mediators may influence redox balance, inflammatory pathways, and cellular metabolism. Despite well-described mechanisms at the experimental level, the translation of these effects into consistent clinical outcomes remains uncertain. Most available clinical data are derived from small, heterogeneous studies, and high-quality randomized controlled trials are limited. Variability in treatment protocols further complicates interpretation and reduces comparability across studies. While potential therapeutic benefits have been reported in selected conditions, including musculoskeletal disorders, wound healing, and supportive care, the overall quality of evidence remains low to moderate. In many cases, findings are inconsistent and do not demonstrate clear superiority over established treatments. Consequently, ozone therapy should be regarded as an adjunctive and investigational approach rather than a validated clinical intervention. A cautious and evidence-based interpretation is required, and further well-designed studies are necessary to clarify its clinical role and establish standardized treatment protocols.

## 15 Conclusion

Medical ozone therapy is a biologically active intervention supported by experimental and translational research, with proposed mechanisms involving redox modulation, immune regulation, and effects on microcirculation. Despite a long history of clinical use and reported benefits in selected conditions, the current clinical evidence remains heterogeneous and limited by variability in study design, small sample sizes, and lack of standardized protocols.

It should be noted that evidence-based medicine includes not only randomized controlled trials but also observational data and clinical expertise. In this context, medical ozone therapy is supported by accumulated clinical experience; however, these findings have not yet been consistently validated in high-quality studies.

When applied at controlled low concentrations and according to established protocols, medical ozone is generally considered safe, although long-term safety data remain limited and outcomes depend on appropriate dosing and administration. Clinical experience suggests that, when properly administered within recommended concentration ranges, medical ozone therapy is associated with a favorable safety profile.

Overall, medical ozone therapy may have a potential adjunctive role in selected clinical settings, but its clinical relevance remains incompletely established. Further well-designed randomized controlled trials and standardized treatment approaches are needed to clarify its therapeutic value and define its role in evidence-informed clinical practice.
